# Inhibitory effect of rape pollen supercritical CO_2_ fluid extract against testosterone-induced benign prostatic hyperplasia in rats

**DOI:** 10.3892/etm.2014.1680

**Published:** 2014-04-14

**Authors:** BI-CHENG YANG, LI-LI JIN, YI-FANG YANG, KUN LI, DAN-MING PENG

**Affiliations:** 1 Department of Traditional Chinese Medicine, Shanghai Institute of Pharmaceutical Industry, Shanghai 200040, P.R. China; 2 Key Laboratory of Women’s Reproductive Health of Jiangxi Province, Jiangxi Maternal and Child Health Hospital, Nanchang, Jiangxi 330006, P.R. China; 3 Jiangxi Institute of Traditional Chinese Medicine, Nanchang, Jiangxi 330000, P.R. China

**Keywords:** rape pollen, benign prostatic hyperplasia, 5α-reductase, cyclooxygenase-2

## Abstract

Benign prostatic hyperplasia (BPH) can lead to lower urinary tract symptoms. Rape pollen is an apicultural product that is composed of nutritionally valuable and biologically active substances. The aim of the present study was to investigate the protective effect of rape pollen supercritical CO_2_ fluid extract (SFE-CO_2_) in BPH development using a testosterone-induced BPH rat model. BPH was induced in the experimental groups by daily subcutaneous injections of testosterone for a period of 30 days. Rape pollen SFE-CO_2_ was administered daily by oral gavage concurrently with the testosterone injections. Animals were sacrificed at the scheduled termination and the prostates were weighed and subjected to histopathological examination. Testosterone, dihydrotestosterone (DHT), 5α-reductase and cyclooxygenase-2 (COX-2) levels were also measured. BPH-induced animals exhibited an increase in prostate weight with increased testosterone, DHT, 5α-reductase and COX-2 expression levels. However, rape pollen SFE-CO_2_ treatment resulted in significant reductions in the prostate index and testosterone, DHT, 5α-reductase and COX-2 levels compared with those in BPH-induced animals. Histopathological examination also demonstrated that rape pollen SFE-CO_2_ treatment suppressed testosterone-induced BPH. These observations indicate that rape pollen SFE-CO_2_ inhibits the development of BPH in rats and these effects are closely associated with reductions in DHT, 5α-reductase and COX-2 levels. Therefore, the results of the present study clearly indicate that rape pollen SFE-CO_2_ extract may be a useful agent in BPH treatment.

## Introduction

Benign prostatic hyperplasia (BPH), a condition characterized by excessive and uncontrolled growth of the prostate gland, affects ~85% of males over 50 years of age (1). Considering the high incidence of BPH and the effect this condition has on the quality of life, treatment of this disease is a priority for public health (2). The aetiology of BPH is complicated and remains unclear; however, recent novel observations highlight the key role of aging (3), hormonal alterations (4), metabolic syndrome (5) and inflammation (6).

At present, pharmacotherapy remains the modality of choice for BPH treatment and may be roughly divided into three groups: α-blockers, 5α-reductase inhibitors and alternative therapies (7). However, these prescription medications may have adverse side-effects, including orthostatic hypotension, decreased libido and ejaculatory or erectile dysfunction (8). Due to these risks, natural products that appear to have limited adverse events are becoming increasingly important in the treatment of BPH (9). Previous studies have shown that a number of natural products, including saw palmetto (10), *Sphaeranthus indicus*, *Pygeum africanum* and *Hypoxis rooperi*, possess anti-BPH potential (11).

Bee-collected pollen is an apicultural product that is composed of nutritionally valuable substances and considerable amounts of biologically active substances (12). Rape (*Brassica campestris* L. var. oleifera DC.) is planted in the majority of regions worldwide. In China, the bee pollen of this plant is widely used as a natural supplement to everyday meals and as an herbal medicine to strengthen the resistance of the body to diseases. This is due to the abundant nutrient properties, including sugars, proteins, lipids, vitamins, carbohydrates and phenolic compounds (13–15). The use of supercritical fluid extracts (SFEs) has been increasingly studied due to their unique properties, versatile applications and changes in environmental regulations that foster the utilization of green solvents. In this field, CO_2_ has been particularly studied since it is essentially non-toxic, non-flammable, inexpensive, recyclable, totally dissipated from extracts at atmospheric pressure and has easily accessible critical conditions. The aims of the present study were to investigate the effects of rape pollen SFE-CO_2_ on testosterone-induced BPH in rats and the underlying molecular mechanism. SFE-CO_2_ was selected since it is known to be rich in fatty acids and their derivatives and steroids. In addition, pollen extract contains a complex mixture of compounds that function in concert to exert a specific bioactivity more effectively than individual compounds.

## Materials and methods

### SFE-CO_2_ extraction

Pollen from *Brassica campestris* L. var. oleifera DC. was collected from Inner Mongolia (China) in July 2008 and was identified by Professor Xu Feng (Jiangsu Institute of Botany, Nanjing, China). A voucher specimen (PN-2008-01) was deposited in the Herbarium of Shanghai Institute of Pharmaceutical Industry (Shanghai, China). Two 1,000-g samples of dried pollen, of which the cell walls were lysed by zymolysis, were extracted by SFE-CO_2_ at 40 MPa and 50°C. The combined extract was evaporated under a reduced pressure to produce a yellow gum (yield, 83 g).

### Animals

Specific pathogen-free male Sprague-Dawley rats with an initial body weight of 230–250 g were purchased from Shanghai Xipuer - Bi Kai Experimental Animals Ltd. (Shanghai, China). The rats were housed in clean pathogen-free rooms in an environment with controlled temperature (22°C), humidity and a 12 h light/dark cycle. Rats had free access to water and a standard laboratory diet. All animal procedures were conducted strictly in accordance with the International Ethical Guidelines and the guide for the Care and Use of Laboratory Animals. Experiments were approved by the Institutional Animal Care and Use Committee of Shanghai Institute of Pharmaceutical Industry.

### Construction of the rat BPH model and drug administration

A rat model of BPH was induced by subcutaneous (sc) injections of testosterone propionate following castration. One week following surgery, the rats were randomly divided into five groups (n=7): Castration (saline 10 ml/kg), model (saline 10 ml/kg), finasteride (5 mg/kg) and two rape pollen SFE-CO_2_ extract groups (21.3 or 88.7 mg/kg). Rats in the model and treatment groups received saline or drug via gastrogavage, in combination with sc injection of 5 mg/kg testosterone propionate daily for 30 days, while those in the castration group received saline by gastrogavage and 1 ml corn oil by sc injection. The body weight of each rat was measured once a week.

Animals were anesthetized with pentobarbital (100 mg/kg body weight; i.p.) following final treatment and overnight fasting. Blood samples were collected from the caudal vena cava. Serum was separated by centrifugation and stored at −80°C. Whole prostates were immediately removed and weighed and relative organ weights were calculated as the ratio of organ weight to body weight. Sections of the ventral prostate lobe were fixed with 10% neutral buffered formalin and embedded in paraffin for histological analysis. The remaining prostate samples were stored at −80°C.

### Prostate index

The prostate index of each rat was the ratio of prostate weight to body weight (mg/g) (16).

### Determination of testosterone and dihydrotestosterone (DHT) levels in the serum and prostate

Prostate tissue was homogenized (1/10, w/v) using a homogenizer in a tissue lysis/extraction reagent containing a protease inhibitor cocktail (Sigma-Aldrich, St. Louis, MO, USA). Homogenates were centrifuged at 12,000 × g for 25 min at 4°C and the protein concentration in the supernatant fractions was determined using a bicinchoninic acid protein assay kit (Pierce Biotechnology, Inc., Rockford, IL, USA), according to the manufacturer’s instructions.

Testosterone and DHT levels in the serum and prostate were measured using an enzyme-linked-immunosorbent assay. DHT and testosterone kits were purchased from Bio-Rad Laboratories, Inc. (Hercules, CA, USA).

### Histopathological examination

To assess morphological changes in the prostate, tissues were embedded in paraffin, cut into sections of 4 μm thickness and stained with hematoxylin and eosin (MHS-16 and HT110-1-32; Sigma-Aldrich). Tissues were subsequently mounted and coverslipped, using mounting medium, for microscopic examination (Nikon, Tokyo, Japan).

### Immunohistochemical detection of 5α-reductase and cyclooxgenase-2 (COX-2)

Paraffin-embedded tissue sections of 3 μm thickness, collected from three rats per group, were deparaffinized with xylene, hydrated using an ethanol series and heated in citrate buffer (pH 6.0) for 5 min. Next, the sections were blocked with 5% bovine serum albumin (BSA) in Tris-buffered saline (TBS) for 2 h. This was followed by incubation at a concentration of 1 μg/ml with anti-5α-reductase or anti-COX-2 rabbit monoclonal antibodies (AbD Serotec, Oxford, UK) with 5% BSA in TBS overnight at 4°C. After washing the slides with TBS, the sections were incubated with the corresponding secondary antibody (Abcam, Cambridge, MA, USA). Sections were then washed with TBS and incubated for 10 min in a solution of 0.02% diaminobenzidine containing 0.01% H_2_O_2_. Counterstaining was performed using hematoxylin and the slides were visualized under a light microscope. At least three sections per rat were investigated and immunohistochemical quantification was conducted using image analysis software (Optimas 6.5, Bothell, WA, USA).

### Statistical analysis

Measurement data are expressed as the mean ± SD. Statistically significant differences between treated and control groups were determined using one-way analysis of variance. P<0.05 was considered to indicate a statistically significant difference. These results were analyzed with SPSS 16.0 statistical software (SPSS Inc., Chicago, IL, USA)

## Results

### Effect of rape pollen SFE-CO_2_ on the prostatic index

Prostatic index is an important indicator in BPH. As shown in Table I, the testosterone-induced BPH group exhibited a significant increase in prostatic index compared with the vehicle-treated group. By contrast, the finasteride-treated group demonstrated a significant reduction in prostatic index compared with the testosterone-induced BPH group. The rape pollen SFE-CO_2_ groups showed significant reductions in prostatic index compared with the testosterone-induced BPH group.

### Effect of rape pollen SFE-CO_2_ on testosterone and DHT levels in the serum

**Figure 1:**
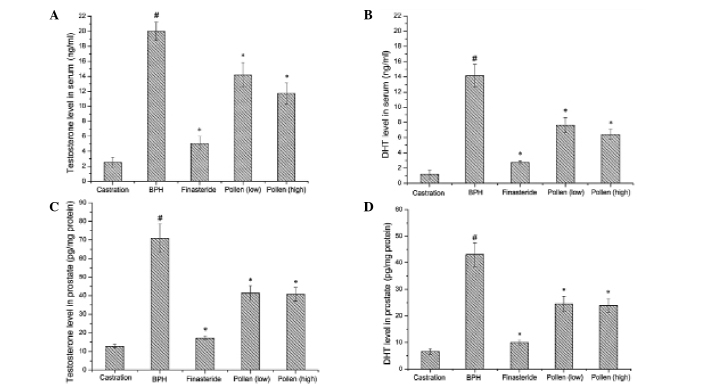
Effects of rape pollen SFE-CO_2_ on (A and C) testosterone and (B and D) DHT levels in (A and B) serum and (C and D) prostate. The rape pollen SFE-CO_2_ treatment groups exhibited significantly decreased testosterone and DHT levels in the serum and prostate compared with the BPH group. Rape pollen SFE-CO_2_ or finasteride treatment was administered 1 h prior to testosterone injection. ^#^P<0.01, vs. castration; ^*^P<0.05, vs. BPH. Castration, corn oil injection (sc) + PBS (p.o.); BPH, testosterone (sc) + PBS (p.o.); finasteride, 5 mg/kg finasteride (p.o.) + testosterone (sc); pollen (low), 21.3 mg/kg rape pollen SFE-CO_2_ (p.o.) + testosterone (sc); pollen (high), 88.7 mg/kg rape pollen SFE-CO_2_ (p.o.) + testosterone (sc); SFE, supercritical fluid extract; DHT, dihydrotestosterone; BPH, benign prostatic hyperplasia; PBS, phosphate-buffered saline.

As shown in Fig. 1A and B, the testosterone-induced BPH group had significantly increased serum testosterone levels compared with those in the castration group. However, the finasteride- and pollen-treated groups had significantly decreased serum testosterone levels compared with those in the testosterone-induced BPH group. Serum DHT levels in the testosterone-induced BPH group were significantly increased compared with those in the castration group. However, the serum DHT levels in the finasteride- and pollen-treated groups were significantly decreased compared with those in the testosterone-induced BPH group.

### Effects of rape pollen SFE-CO_2_ on testosterone and DHT levels in the prostate

In the prostate, while the testosterone-induced BPH group exhibited increased levels of testosterone and DHT compared with those in the castration group, the finasteride-treated group had markedly decreased testosterone and DHT levels compared with those in the BPH group. Similarly, the pollen-treated group exhibited significantly reduced testosterone and DHT levels compared with those in the BPH group (Fig. 1C and D).

### Effect of rape pollen SFE-CO_2_ on prostate tissue by histopathological examination

**Figure 2:**
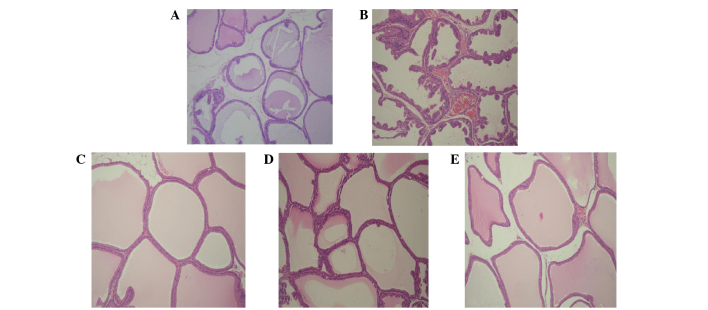
Effects of rape pollen SFE-CO_2_ on prostate hyperplasia in (A) castration, (B) BPH, (C) finasteride, (D) pollen (low) and (E) pollen (high) groups. Histological examination of the prostate tissue was performed 24 h after the final testosterone injection. Prostate tissues were fixed, sectioned at 4 μm thickness and stained with hematoxylin and eosin solution (magnification, ×1,200). Castration, corn oil injection (sc) + PBS (p.o.); BPH, testosterone (sc) + PBS (p.o.); finasteride, 5 mg/kg finasteride (p.o.) + testosterone (sc); pollen (low), 21.3 mg/kg rape pollen SFE-CO_2_ (p.o.) + testosterone (sc); pollen (high), 88.7 mg/kg rape pollen SFE-CO_2_ (p.o.) + testosterone (sc); SFE, supercritical fluid extract; BPH, benign prostatic hyperplasia; PBS, phosphate-buffered saline.

As shown in Fig. 2, epithelial cell layers and stromal spaces in the prostate were larger in the testosterone-induced BPH group compared with those in the castration group. The finasteride-treated group exhibited mild glandular hyperplasia compared with the testosterone-induced BPH group. Pollen-treated animals also exhibited a reduction in epithelial cell layers and stromal spaces compared with the BPH group, which was similar to the finasteride-treated group.

### Effect of rape pollen SFE-CO_2_ on 5α-reductase expression

**Figure 3:**
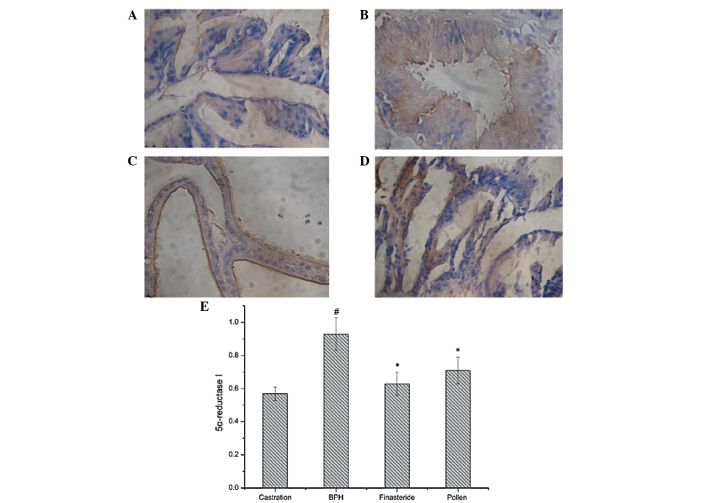
Immunohistochemical staining of 5α-reductase type I in the prostate tissues of rats in the (A) castration, (B) BPH, (C) finasteride and (D) pollen groups. (E) Quantitative image analysis of the immunohistochemical staining expressed as optical densities across 10 fields for each rat section. ^#^P<0.05, vs. castration; ^*^P<0.05, vs. BPH. Castration, corn oil injection (sc) + PBS (p.o.); BPH, testosterone (sc) + PBS (p.o.); finasteride, 5 mg/kg finasteride (p.o.) + testosterone (sc); pollen, 88.7 mg/kg rape pollen SFE-CO_2_ (p.o.) + testosterone (sc); SFE, supercritical fluid extract; BPH, benign prostatic hyperplasia; PBS, phosphate-buffered saline. Magnification, ×100.

**Figure 4:**
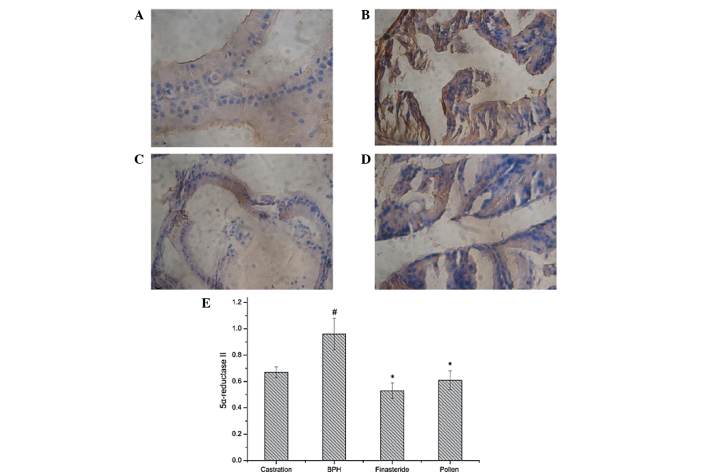
Immunohistochemical staining of 5α-reductase type II in the prostate tissues of rats in the (A) castration, (B) BPH, (C) finasteride and (D) pollen groups. (E) Quantitative image analysis of the immunohistochemical staining expressed as optical densities across 10 fields for each rat section. ^#^P<0.05, vs. castration; ^*^P<0.05, vs. BPH. Castration, corn oil injection (sc) + PBS (p.o.); BPH, testosterone (sc) + PBS (p.o.); finasteride, 5 mg/kg finasteride (p.o.) + testosterone (sc); pollen, 88.7 mg/kg, rape pollen SFE-CO_2_ (p.o.) + testosterone (sc); SFE, supercritical fluid extract; BPH, benign prostatic hyperplasia; PBS, phosphate-buffered saline. Magnification, ×100.

Expression levels of 5α-reductase I and II were detected immunohistochemically. As shown in Figs. 3 and 4, the testosterone-induced BPH group had significantly increased 5α-reductase I and II expression levels compared with those in the castration group. However, the finasteride- and pollen-treated groups had significantly decreased 5α-reductase I and II expression levels compared with those in the testosterone-induced BPH group.

### Effect of rape pollen SFE-CO_2_ on COX-2 expression

**Figure 5:**
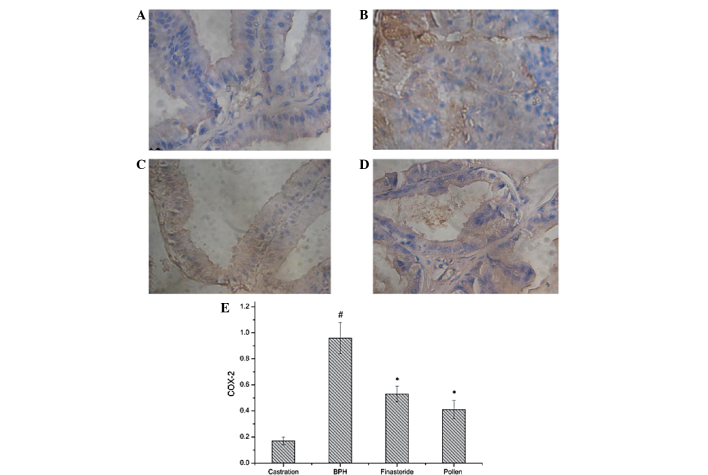
Immunohistochemical staining of COX-2 in the prostate tissues of rats in the (A) castration, (B) BPH, (C) finasteride and (D) pollen groups. (E) Quantitative image analysis of the immunohistochemical staining expressed as optical densities across 10 fields for each rat section. ^#^P<0.05, vs. castration; ^*^P<0.05, vs. BPH. Castration, corn oil injection (sc) + PBS (p.o.); BPH, testosterone (sc) + PBS (p.o.); finasteride, 5 mg/kg finasteride (p.o.) + testosterone (sc); pollen, 88.7 mg/kg rape pollen SFE-CO_2_ (p.o.) + testosterone (sc); SFE, supercritical fluid extract; BPH, benign prostatic hyperplasia; PBS, phosphate-buffered saline; COX-2, cyclooxygenase-2. Magnification, ×100.

As shown in Fig. 5, COX-2 expression levels in the testosterone-induced BPH group significantly increased compared with those in the castration group. However, COX-2 levels in the finasteride- and pollen-treated groups were significantly decreased compared with those in the testosterone-induced BPH group.

## Discussion

In the present study, the effects of rape pollen SFE-CO_2_ on prostate size and DHT and testosterone levels were evaluated in the prostate tissue and serum of a testosterone-induced BPH rat model. Testosterone-induced rats exhibited increases in prostate size, DHT levels and 5α-reductase and COX-2 expression levels when compared with the castration group. In addition, prostatic hyperplasia was observed during histopathological examinations. However, rape pollen SFE-CO_2_-treated rats exhibited reductions in prostate size, levels of DHT and testosterone in the serum and prostate and expression levels of 5α-reductase and COX-2 in the prostate when compared with testosterone-induced rats. Histopathological examination also demonstrated that oral administration of rape pollen SFE-CO_2_ attenuated testosterone-induced prostatic hyperplasia.

Rats with BPH demonstrated significant increases in prostatic index compared with the negative control animals. However, pollen-treated animals exhibited significant reductions in these measures when compared with the BPH animals. According to previous studies, increased prostatic index is an important marker indicating the development of BPH (17,18). BPH involves epithelial and stromal hyperplasia of the prostate (19,20), resulting in an increase in prostate weight. When sufficiently large, the prostate constricts the urethral canal to cause partial, or in certain cases, complete obstruction (21). For these reasons, a number of studies have investigated the inhibitory effects of various substances on the development of BPH by measuring the prostatic index (22). The results of the present study indicate that rape pollen SFE-CO_2_ administration causes a significant reduction in the prostatic index when compared with the testosterone-induced BPH group. These results were consistent with the histopathological examinations of the prostate tissues. BPH animals experienced stromal proliferation and glandular hyperplasia in the prostate, whereas animals treated with rape pollen SFE-CO_2_ exhibited mild glandular hyperplasia. These observations indicate that rape pollen SFE-CO_2_ is an effective treatment for BPH.

The genesis of BPH depends on two factors: Testicular androgen and the aging process (23). The most important androgen in the prostate is DHT (24). DHT is formed through the reduction of testosterone, catalyzed by the enzyme 5α-reductase. This enzyme has two isoenzymes: 5α-reductase type I and II (25). Dysregulation of the reaction converting testosterone to DHT by 5α-reductase has been reported to be a key step in the development of BPH. In addition, elevated DHT levels correlate with the pathogenesis and progression of androgen-dependent diseases, including prostate cancer and BPH (26). BPH has been successfully treated with 5α-reductase inhibitors that lower the level of DHT available to the prostate tissue by blocking the action of 5α-reductase that converts testosterone into DHT. A number of studies have been conducted with the aim of reducing DHT levels via the inhibition of 5α-reductase. Finasteride is a 5α-reductase inhibitor and an elective drug used for the treatment of BPH. Finasteride reduces testosterone and DHT levels in the serum and prostate, resulting in a reduction in prostate size and ultimately providing relief from the lower urinary tract symptoms associated with BPH (22). However, finasteride also produces serious side-effects (27), which has led to a number of studies investigating alternative materials for treating BPH with fewer side-effects (28). Natural products that appear to have limited adverse events are becoming increasingly important in the treatment of BPH. Previous studies have shown that numerous natural products, including saw palmetto (10) *Sphaeranthus indicus*, *Pygeum africanum* and *Hypoxis rooperi*, possess anti-BPH potential (29). The present study identified that finasteride reduced testosterone and DHT levels in the serum and prostate, as well as the 5α-reductase expression levels in the prostate. In addition, rape pollen SFE-CO_2_ decreased the levels of testosterone and DHT in the serum and prostate and also significantly decreased 5α-reductase I and II expression compared with that in the testosterone-induced BPH group. These observations indicate that rape pollen SFE-CO_2_ inhibits the development of BPH in rats and these effects were closely associated with a reduction in 5α-reductase expression levels.

COX-2 is a proinflammatory inducible enzyme whose production is triggered by mitogens, cytokines, reactive oxygen species and growth factors in a variety of cell types. Increased mRNA expression levels of COX-2 have been documented in BPH, particularly in luminal epithelial cells (30). Several mechanisms have been proposed to explain the role of COX-2 in prostate overgrowth. Certain effects may result from COX-2-mediated increases in prostaglandin (PG) synthesis, particularly PGE2 (31). However, COX-2 also upregulates antiapoptotic protein Bcl-2 expression with a concomitant decrease in prostate tissue apoptosis (32). Previous observations have indicated that two COX-2 selective inhibitors, rofecoxib and celecoxib, are effective as monotherapy or in combination with finasteride for the management of lower urinary tract symptoms in human BPH (33,34). The present study found that COX-2 levels in the pollen-treated group significantly decreased compared with those in the testosterone-induced BPH group. These observations indicate that rape pollen SFE-CO_2_ inhibits the development of BPH in rats and these effects were closely associated with a reduction in COX-2 expression.

In conclusion, oral administration of rape pollen SFE-CO_2_ in a BPH rat model significantly decreased the prostatic index, as well as the DHT, 5α-reductase and COX-2 expression levels. These observations indicate that rape pollen SFE-CO_2_ inhibits the development of BPH in rats and these effects were closely associated with a reduction in the levels of DHT, 5α-reductase and COX-2. The results of the present study clearly indicate that rape pollen SFE-CO_2_ may be useful in BPH treatment.

## Figures and Tables

**Table I:** Effect of rape pollen extract on the prostatic index.

Group	Treatment	Prostatic index ×10^−3^
Castration	Vehicle-treated	2.124±0.075
BPH model	Testosterone	4.166±0.070^a^
Finasteride	Testosterone + 5 mg/kg finasteride	3.287±0.122^b^
Pollen extract (low)	Testosterone + 21.3 mg/kg pollen extract	3.890±0.103^b^
Pollen extract (high)	Testosterone + 88.7 mg/kg pollen extract	3.469±0.144^b^

Prostate index is the ratio of prostate weight to body weight (mg/g). Values are expressed as mean ± SD and data were analyzed by one-way analysis of variance.

a P<0.05, vs. castration control;

b P<0.05, vs. BPH model.
